# Leveraging Machine Learning to Develop Digital Engagement Phenotypes of Users in a Digital Diabetes Prevention Program: Evaluation Study

**DOI:** 10.2196/47122

**Published:** 2024-03-01

**Authors:** Danissa V Rodriguez, Ji Chen, Ratnalekha V N Viswanadham, Katharine Lawrence, Devin Mann

**Affiliations:** 1 New York University Grosman School of Medicine New York, NY United States; 2 New York University Langone Health New York, NY United States

**Keywords:** machine learning, digital health, diabetes, mobile health, messaging platforms, user engagement, patient behavior, digital diabetes prevention programs, digital phenotypes, digital prescription, users, prevention, evaluation study, communication, support, engagement, phenotypes, digital health intervention, chronic disease management

## Abstract

**Background:**

Digital diabetes prevention programs (dDPPs) are effective “digital prescriptions” but have high attrition rates and program noncompletion. To address this, we developed a personalized automatic messaging system (PAMS) that leverages SMS text messaging and data integration into clinical workflows to increase dDPP engagement via enhanced patient-provider communication. Preliminary data showed positive results. However, further investigation is needed to determine how to optimize the tailoring of support technology such as PAMS based on a user’s preferences to boost their dDPP engagement.

**Objective:**

This study evaluates leveraging machine learning (ML) to develop digital engagement phenotypes of dDPP users and assess ML’s accuracy in predicting engagement with dDPP activities. This research will be used in a PAMS optimization process to improve PAMS personalization by incorporating engagement prediction and digital phenotyping. This study aims (1) to prove the feasibility of using dDPP user-collected data to build an ML model that predicts engagement and contributes to identifying digital engagement phenotypes, (2) to describe methods for developing ML models with dDPP data sets and present preliminary results, and (3) to present preliminary data on user profiling based on ML model outputs.

**Methods:**

Using the gradient-boosted forest model, we predicted engagement in 4 dDPP individual activities (physical activity, lessons, social activity, and weigh-ins) and general activity (engagement in any activity) based on previous short- and long-term activity in the app. The area under the receiver operating characteristic curve, the area under the precision-recall curve, and the Brier score metrics determined the performance of the model. Shapley values reflected the feature importance of the models and determined what variables informed user profiling through latent profile analysis.

**Results:**

We developed 2 models using weekly and daily DPP data sets (328,821 and 704,242 records, respectively), which yielded predictive accuracies above 90%. Although both models were highly accurate, the daily model better fitted our research plan because it predicted daily changes in individual activities, which was crucial for creating the “digital phenotypes.” To better understand the variables contributing to the model predictor, we calculated the Shapley values for both models to identify the features with the highest contribution to model fit; engagement with any activity in the dDPP in the last 7 days had the most predictive power. We profiled users with latent profile analysis after 2 weeks of engagement (Bayesian information criterion=−3222.46) with the dDPP and identified 6 profiles of users, including those with high engagement, minimal engagement, and attrition.

**Conclusions:**

Preliminary results demonstrate that applying ML methods with predicting power is an acceptable mechanism to tailor and optimize messaging interventions to support patient engagement and adherence to digital prescriptions. The results enable future optimization of our existing messaging platform and expansion of this methodology to other clinical domains.

**Trial Registration:**

ClinicalTrials.gov NCT04773834; https://www.clinicaltrials.gov/ct2/show/NCT04773834

**International Registered Report Identifier (IRRID):**

RR2-10.2196/26750

## Introduction

Over 80 million US adults have prediabetes, a metabolic condition that places individuals at risk for progression to type 2 diabetes and its related complications [1]. Evidence-based strategies for diabetes prevention have primarily focused on nonpharmacologic interventions such as diabetes prevention programs (DPPs), which are comprehensive behavior change curricula concentrating on physical activity and dietary modification. Such programs can be as effective as medication in preventing the progression of diabetes in at-risk populations [2]. Increasingly, DPP behavioral curricula have been adapted to digital platforms (digital DPPs [dDPPs]), which have demonstrated comparable effectiveness in achieving weight loss, hemoglobin A_1c_ reduction, and other critical diabetes-related health outcomes while offering improvements in accessibility, convenience, and personalization [3]. Yet, limited patient engagement with digital interventions presents a significant barrier to translating evidence-based digital behavioral interventions such as the dDPP into pragmatic, scalable solutions [4-8].

To address this critical patient engagement issue, various technologies and interventions have been developed to provide targeted support to patients using digital health apps to improve engagement and sustained use [9]. Potential solutions include mobile-based feedback and reminder tools, app-based coaching, social networking, and gamification. More recent strategies have also leveraged machine learning (ML) and big data analytics to deploy more advanced tools, such as engagement algorithms and artificial intelligence (AI)–driven chatbots. ML solutions can provide (1) more nuanced patient segmentation or phenotyping; (2) more precise, tailored interventions, with enhanced ability to respond dynamically to changes in individual trends; and (3) improved resource alignment by intervention implementers, as automated processes (eg, chatbots) can free up human capital for more appropriate tasks [10]. Moreover, AI-driven chatbots (AI chatbots), conversational agents that mimic human interaction through written, oral, and visual communication channels with a user [1,2], have demonstrated efficacy in health-behavior change interventions among a large and diverse population [3-6,11-13].

Prior work from this team involved developing a personalized automatic messaging system (PAMS) that leveraged an evidence-based engagement algorithm to deliver tailored behavior change theory–supported SMS text messaging to support users engaging with a commercial app-based dDPP. The study returned promising results compared with average users, demonstrating engagement in various dDPP features (eg, weight tracking and physical activity logins) [12]. To expand on the previous investigation, improved features of the next generation of PAMS include an ML-based patient engagement prediction algorithm to identify dDPP digital engagement phenotypes and to guide and further personalize the messaging intervention. This paper describes the ML model designed to predict characteristics and behavioral patterns of dDPP user types (eg, those highly engaged with exercise but not uploading the meals or those messaging their coach but not participating in weigh-ins) based on their activity patterns within a dDPP app, with a particular focus on motivating users at risk for low engagement and nonengagement with the dDPP (ie, patient digital engagement phenotypes).

## Methods

### Overview

**Figure 1:**
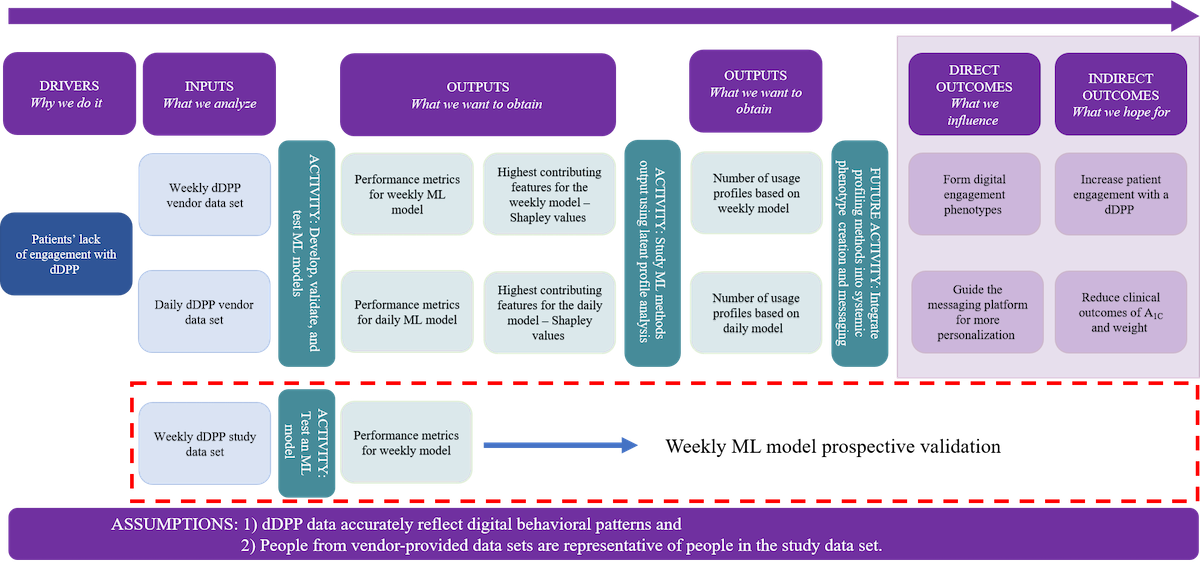
Logic diagram of the research methodology to integrate machine learning (ML) into participant profiling, including the input data sets; the methods applied to the data sets; and the intermediary, direct, and indirect outputs. dDPP: digital diabetes prevention program.

The logic diagram in Figure 1 illustrates, from left to right, the overall framework for optimizing patient engagement with a dDPP [14]. In this study, we completed 2 activities (developing, validating, and testing ML models and studying model outputs with latent profile analysis [LPA]) and identified future activities toward optimization. The drivers behind this optimization initiative stem from low levels of patient engagement with dDPPs and other wellness-based mobile apps. We used the daily and weekly data sets provided by the dDPP vendor (inputs) to develop, validate, and test an ML model for each data set (first activity). On the basis of the performance metrics from the daily and weekly models, we identified the highest contributing feature for each model using Shapley values (first outputs). These features were fed into the LPA (second activity) to determine the number of participant usage profiles (second outputs). The goodness of fit derived from the LPA validated the phenotypes formed from the LPA (direct outcome). This integration of ML and statistical learning processes would inform how we identify digital engagement phenotypes for the dDPP study set (in the dashed red box) and, therefore, design content for a more personalized messaging platform (second direct outcome). Ultimately, the desired long-term outcomes of the profiling process are increased patient engagement with the dDPP and a reduction in clinical outcomes related to hemoglobin A_1C_ and weight (indirect outcomes). The process rests on the assumptions that the dDPP data accurately reflect digital behavioral patterns and that people from the vendor-provided data are representative of people in the study data set.

### Participants

Study participants were users with prediabetes who enrolled in a commercial dDPP app (our dDPP research vendor), including nonpatient (“vendor”) users and institution-based patients (“study” participants of this dDPP intervention) [11]. Eligible participants are at least 18 years old, have a BMI of at least 25 kg/m^2^ (22 kg/m^2^ if self-identified as Asian), have a diagnosis of prediabetes (either by *International Classification of Diseases, Tenth Revision* code, problem list, or a hemoglobin A_1c_ level of 5.7%-6.4% in the last 12 months), and are deemed safe to engage in light physical exercise and weight loss by their primary care physician. For institutional study participants enrolled in the current clinical trial of this dDPP intervention, patients are excluded if they have a prior diagnosis of diabetes, have any end-stage illness with a prognosis within 6 months, are non-English speakers (as the dDPP program is currently only available in English), or are unable to send or receive SMS text messages [4]. Recruited patients were identified via electronic health record review and contacted through multichannel methods (eg, patient portal, email, in-clinic recruitment, and clinician referral).

### The Data

#### Data Sourcing

Data for the evaluation were sourced from a commercial dDPP vendor and a patient cohort of an academic health center. We used 2 deidentified data sets (weekly and daily data) of eligible retail users for the initial training, validation, and testing of the ML models. These data sets aggregate and present user information on a weekly or daily basis and capture all features recorded by the dDPP app, including per user or patient: meals logged, steps logged, exercises logged, messages shared with the dDPP coach and other dDPP patients using the app, app log-ins, and the number of dDPP articles read. These activities were the same as those used for generating the adherence algorithm in our previous research. In addition to the vendor-provided data sets, for a later testing phase, we use an existing data set of data collected from dDPP patients who are part of this dDPP study and exposed to the PAMS intervention.

#### Weekly dDPP Vendor Data Set

Data include detailed information about all the features collected for our dDPP app partners, such as meals logged, steps logged, exercises logged, messages shared with the dDPP coach and other dDPP patients using the app, app log-ins, and the number of dDPP articles read during each week. All users have more than 5 weeks of engagement records, and we used only 1 year’s worth of dDPP engagement data per user.

#### Weekly dDPP Institutional Study Data Set

The 2 data sets (weekly dDPP vendor data set and weekly dDPP study data set) have the same data structure. The same data fields are collected for commercial users and the dDPP patients, but the only difference is on the behavioral level because the patients’ data are potentially affected by the message intervention (PAMS). All data were used for the validation of the weekly ML model.

#### Daily dDPP Vendor Data Set

In addition to the activity records in the weekly data, we had access within the daily data set to calorie consumption data, meal logs, and color codes assigned to each food item as reported by the users. Users with less than 7 days of engagement records were excluded from the cohort, and we used only 1 year’s worth of dDPP engagement data per user.

### Outcomes

First, we built binary classification ML models to predict whether a participant will engage in the next week or the next day with the dDPP based on their previous short- and long-term activity in the app. For the weekly model, we used the vendor data set to train and validate retrospectively to predict general activity (engagement in any activity). We prospectively validated the weekly model using the institutional study data set. For the daily models, we predicted 5 outcomes: general activity, physical activities (steps and exercises recorded on the app), in-app lessons (article reading), social activities (group posts and coach messages in the app), and weigh-ins in the app. Second, we identified the variables from the daily overall activity model of the vendor’s participants that provide the most predictive power for engagement. Third, we evaluated whether these predictive variables could generate profiles of a participant’s behavior that can be targeted with motivational messaging.

### Predictors

We built model predictors from users’ demographic data and collected in-app activities. These activities include steps taken, exercises, meal logs, weigh-in records, in-app messaging and group activities, and in-app article reading. For the weekly data set, short-term activity profiles were built from the week before the evaluation week and up to 4 weeks before the evaluation week. Long-term activity profiles were summarized and constructed from the first week of program enrollment up to the evaluation week. Short-term activity profiles were built from the day before and within 7 days before the evaluation day for the daily data set. Similarly, long-term activity profiles were summarized and constructed from the first day of program enrollment up to the evaluation day. The day of the week and national holidays were also captured as predictors. In total, 43 predictors were used to build weekly models, and 49 predictors were used to build daily models.

### Sample Size

The sample sizes for user weekly and daily data sets directly from the dDPP vendor were determined by the convenience of the dDPP vendor and assumed to be representative of the academic health center’s study sample. The study sample size was determined by the number of participants already recruited and actively involved in the original dDPP study as of December 2021 [4].

### Missing Data

Because this paper aims to predict participant engagement with the dDPP, missing data among in-app activities were treated as a participant not engaging in either overall activity (ie, no observations for a particular day or a week for any activity) or specific within-dDPP activities (eg, a participant not recording meals or reading any articles). Missing participant weight was logged as a participant not weighing themselves for the dDPP, and we ignored the magnitude of weight due to individual non-dDPP factors contributing to weight outcomes. No participant had a missing age due to age being a requirement for enrollment into the dDPP. Participants who did not record their ideal body weight at the beginning of dDPP engagement had this observation recorded as a 0, as the lack of goal recording for weight could have clinical implications (eg, weight is not the primary utilization goal for the participant, or the participant is not comfortable with setting a weight goal). No participant had a missing initial BMI recorded. One participant was missing gender identification, so their observations were removed from the data set.

### Statistical Analysis Methods

#### Data Split

All data sets were split into a 70% training set, a 15% validation set, and a 15% test set based on users. Observations of any user only existed in 1 set to prevent potential data leak and unintended bias.

#### Gradient-Boosted Forest Algorithm

We use the gradient-boosted forest algorithm, a robust regression tree approach that includes multiple simple decision trees to iteratively refine the performance of the model by minimizing the difference between the expected and expert-labeled outcomes [15,16]. Forest-based algorithms provide 2 fundamental benefits. First, they allow for nonlinear interactions between covariates to impact the prediction of the dependent variable, as opposed to using a Least Absolute Shrinkage and Selection Operator (LASSO) or a ridge regression model. Second, forest-based algorithms do not require a priori function structure to define the relationship between the covariates and the outcome. For example, we do not need to theoretically assume whether a particular engagement type (eg, steps) interacts with another type (eg, exercise logging). We used gradient boosting to allow for prediction despite the sparsity of the data, as users may engage with one activity but not others on a given day or have no activity (ie, all observations as 0). The values defining engagement included binary predictors, large integers (eg, calories and steps), and values between 0 and 1 (eg, the portion of engagement throughout enrollment). These models aimed to identify that the sub-behaviors that create the most predictive power for engagement with the dDPP were trained with η=0.1 for 1000 rounds with early stopping.

#### Metrics

The area under the receiver operating characteristic curve (AUROC), the area under the precision-recall curve (AUPRC), and the Brier score statistics measured the performance of the model. To estimate the CIs of the evaluation metrics for the ML models, we performed bootstrapping with 200 iterations on the test set. In each iteration, a random sample of the test set, with replacement, was drawn with the same size as the original test set. The ML model was then evaluated on this bootstrapped sample, and the performance metrics mentioned above were recorded. The process was repeated for 200 iterations, resulting in a distribution of performance metrics from which the 95% CIs were calculated, providing a robust estimate of the performance and variability of the model. In addition, Shapley values were calculated to reflect the feature importance of each model.

#### Engagement Profiling

A person-centered approach to messaging can help motivate individuals to complete goal-oriented behaviors like engagement with a lifestyle management app [17]. This approach involves (1) tailoring delivery based on the person’s behavior profile within the app and (2) focusing messaging on targetable behaviors to motivate users to complete small, manageable actions toward their goal (ie, the goal gradient hypothesis in decision-making) [18]. We performed an LPA on the participants in the daily data set to determine the subgroups of participants’ behaviors. LPA identifies latent clusters of individuals based on continuous variables [19]. The contributions of multiple variables (ie, the facets that explain the unobserved profile of a user) contribute to the outcome experienced by a user. We used the covariates with the highest global mean Shapley values from the gradient-boosted forest model for the LPA for 2 reasons. First, these variables offer the most explanatory power behind the probability of engagement with the dDPP, allowing us not to assume a priori the behaviors that contribute to the usage of the dDPP. Second, profiling users of a digital app such as this dDPP can be more complicated than traditional approaches to consumer profiling, given the interaction between a user’s health and app engagement. To determine the minimum usage data after enrollment into a dDPP to start profiling participants, we conducted LPAs after 2 weeks and iteratively added days until 3 weeks of engagement. We used the profiles from the timestamp with the lowest Bayesian information criterion (BIC), the established goodness-of-fit metric for LPA. We used the *mclust* package in RStudio (version 2022.12.0+353; Posit Software, PBC) to run the LPAs [20].

#### Development Versus Validation

We validated the weekly model prospectively using the weekly dDPP study data set. Detailed information about this data set is under the subsection “Participants” [15,16].

### Ethical Considerations

In this DPP research, ethical standards and the protection of human participants are emphasized. The study is committed to adhering to regulations outlined in 45 CFR Part 46, ensuring the rights and welfare of participants. The NYU Langone Health institutional review board (IRB) played a crucial role in reviewing and approving the research, informed consent forms, and recruitment materials before participant enrollment (i20-01548). The informed consent process is described as an ongoing dialogue, emphasizing clear communication, comprehension, and the right to withdraw without adverse consequences. The consent forms, including verbal consent and a key information sheet, were submitted to the IRB for approval. Confidentiality measures are robust, complying with the Health Insurance Portability and Accountability Act (HIPAA), and a Certificate of Confidentiality from the National Institutes of Health was obtained. Data security is maintained through password protection, and research data are stored securely. The research emphasizes that stored data will only be used for this study, with no plans for future use in subsequent research. Overall, the research underscores the importance of ethical conduct, participant consent, and stringent confidentiality measures in the research process.

Moreover, the research underscores the importance of ethical conduct, rigorous IRB oversight, and robust confidentiality measures to safeguard the rights and well-being of study participants. Additionally, it highlights the meticulous documentation of the informed consent process and the secure handling of research data, ensuring compliance with regulations and promoting participant trust and privacy.

## Results

### Participants

Table 1 details the descriptive statistics for the 3 preprocessed data sets, including weekly and daily data for the dDPP user (dDPP vendor data sets) and the weekly data for the dDPP patients (dDPP study data). For the vendor-provided data sets, users engage with the app 54.2% (208,142/384,025) of the times in the weekly data compared with 38.9% (274,200/704,242) of the times in the daily data. The average engagement within individual activities is similar. “Steps taken” had the highest percentage of all activities in both data sets. For study data, the engagement percentage was higher (92.1%, 1253/1361), which could be attributed to the effects of PAMS messages.

**Table 1:** Descriptive statistics of users (N=12,262).

Characteristic			Weekly dDPP^a^ vendor data (dDPP vendor users, n=10,053)		Weekly dDPP study data (dDPP study patients, n=50)		Daily dDPP vendor data (dDPP vendor users, n=2159)
Program length			38.2 weeks		27.22 weeks		326.2 days
Age (years), mean (SD)			47.6 (11.4)		N/A^b^		N/A
**Sex, n (%)**							
	Male	1267 (12.6)		N/A		N/A	
	Female	8786 (87.4)		N/A		N/A	
Engagement of any activity, n/N (%)			208,142/384,025 (54.2)		1253/1361 (92.1)		274,200/704,242 (38.9)
Engagement of steps taken, n/N (%)			208,142/384,025 (54.2)		1086/1361 (79.8)		244,823/704,242 (34.8)
Engagement of exercises, n/N (%)			77,957/384,025 (20.3)		349/1361 (25.6)		49,683/704,242 (7.1)
Engagement of meals logged, n/N (%)			137,865/384,025 (35.9)		924/1361 (67.9)		100,449/704,242 (14.3)
Engagement of weigh-ins, n/N (%)			137,481/384,025 (35.8)		523/1361 (38.4)		71,596/704,242 (10.2)
Engagement of article reading, n/N (%)			118,280/384,025 (30.8)		573/1361 (42.1)		79,272/704,242 (11.2)
Engagement of group posts, n/N (%)			24,578/384,025 (6.4)		100/1361 (7.3)		45,113/704,242 (6.4)

^a^dDPP: digital diabetes prevention program.

^b^N/A: not applicable.

### Weekly Model (for Any Activity) Development and Performance

**Figure 2:**
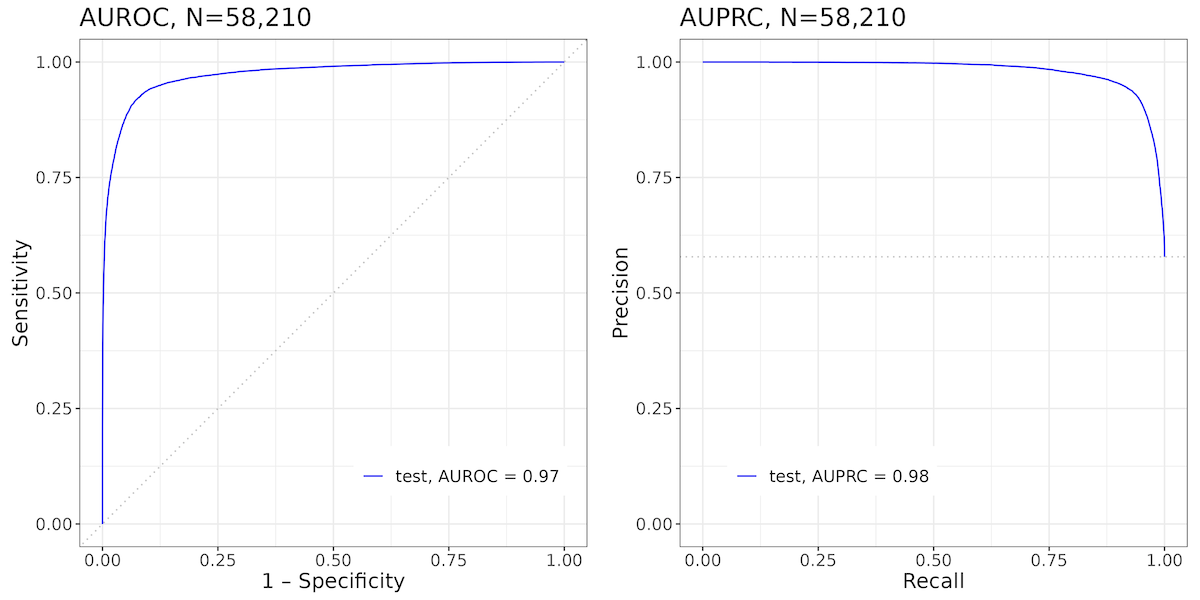
AUROC (left) and AUPRC (right) performance metrics of the “any activity” weekly model in the test set of the weekly vendor data set (58,210 engagement records). The calibration plot shows that the model is well calibrated. AUPRC: area under the precision-recall curve; AUROC: area under the receiver operating characteristic curve.

We trained and tested the model to predict “any activity” (ie, the probability of the subsequent interaction with the dDPP based on whether the user interacted with any of the features of the dDPP app, such as exercise, meal, and weigh-ins) on the weekly dDPP vendor data set. The weekly model reported an AUROC of 0.97 (95% CI 0.97-0.97), an AUPRC of 0.98 (95% CI 0.98-0.98), and a Brier score of 0.061 (95% CI 0.060-0.063) in the test set (Figure 2). Because we also aimed to identify how individual variables contribute to predictions by the model, we calculated the Shapley value, which is the average marginal contribution of a variable to a model across the different combinations of including the variable in the model (eg, nonlinear contributions and splitting a forest into different branches with the variable). The Shapley value method has become the preferred technique for feature attribution in ML models, thanks to its robust and reliable performance [21].

**Figure 3:**
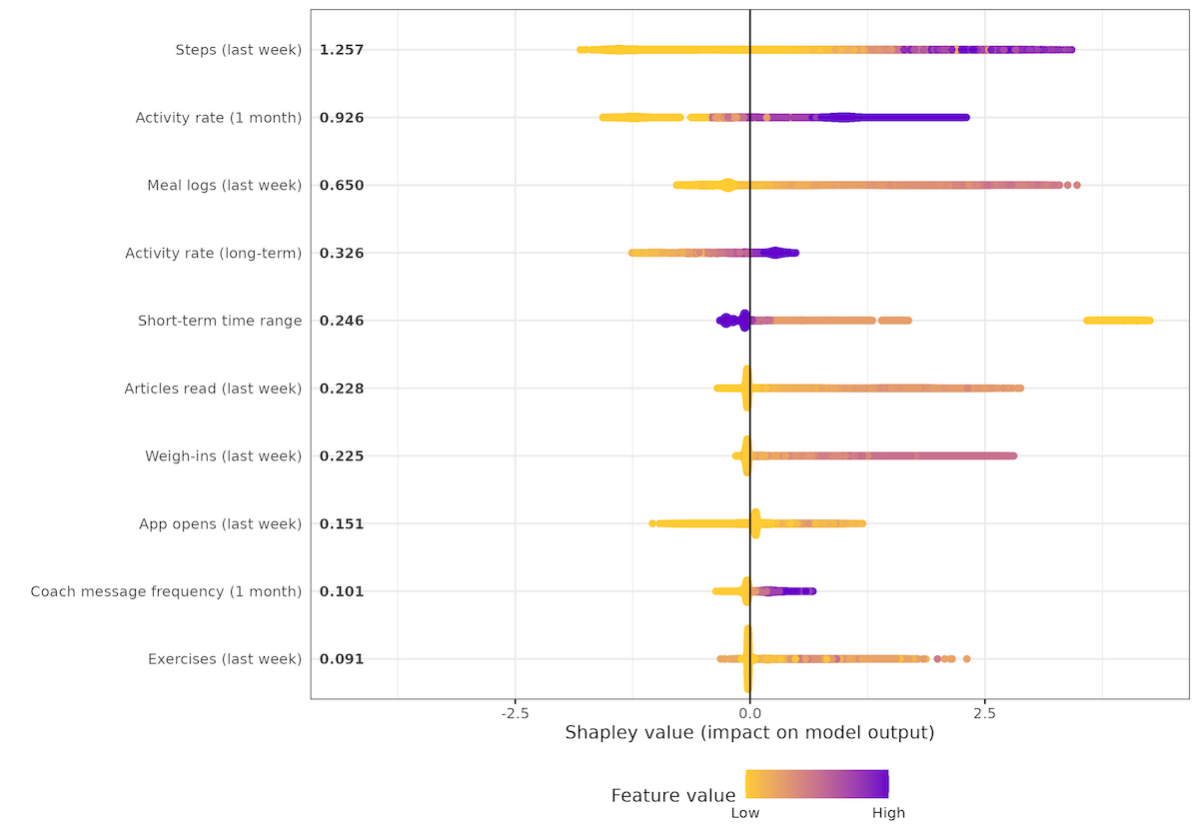
Shapley values of top 10 features in the “any activity weekly model.” Each dot on the plot represents an engagement record and is colored according to the value of the corresponding feature from high (purple) to low (yellow). Features are ranked in descending order from top to bottom on the y-axis (ie, variables with the highest contribution to the model are on the top), with global mean Shapley values of each feature annotated next to them.

Figure 3 displays the distribution of the 10 covariates with the highest calculated global mean Shapley value (ie, which variables have the strongest predictive power, regardless of negative or positive impact, on the user’s engagement with the dDPP). A higher magnitude of the Shapley value (ie, further from 0) indicates the strength of the variable in the model to predict a user’s engagement with the dDPP. A positive Shapley value indicates that the user is more likely to engage with the dDPP because of the variable (ie, a positive predictor). A negative Shapley value suggests that the patient is less likely to engage with the dDPP due to the variable (ie, a negative predictor). More purple values indicate a higher mean for the covariate of the individual (eg, a more purple “exercise frequency” dot indicates that the user logged for nonstep physical activity more than other users did). The covariates with the most contribution to model prediction were those of short-term behaviors.

**Figure 4:**
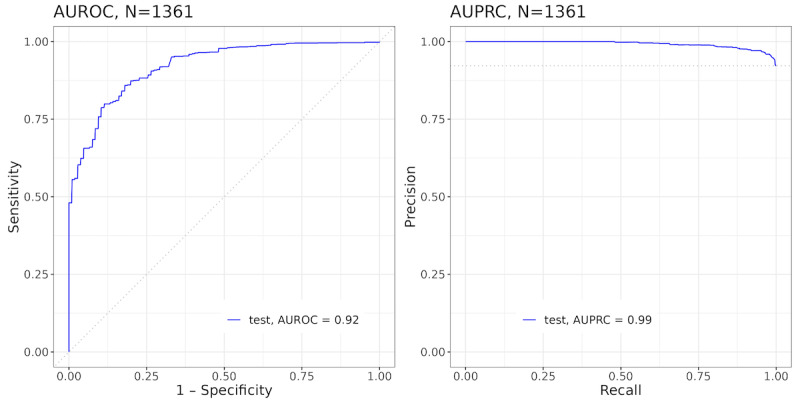
AUROC (left) and AUPRC (right) performance metrics of any activity weekly model in the weekly study data set (1361 engagement records). AUPRC: area under the precision-recall curve; AUROC: area under the receiver operating characteristic curve.

We tested our model using the weekly dDPP institutional study data set (prospective clinical data). The model achieved an AUROC of 0.92 (95% CI 0.89-0.94), an AUPRC of 0.99 (95% CI 0.99-0.99; Figure 4), and a Brier score of 0.072 (95% CI 0.063-0.081), suggesting high predictive power and operational potential for refining PAMS using this method. After analyzing the weekly dDPP study data set, we detected that this data set would be imbalanced because the prediction of the subsequent week’s activity would be based on whether a user engaged with any app activity, rather than a particular activity, within the dDPP, seen by the 92.1% engagement ratio, and the sample size was too low to yield unbiased testing results. Regardless of the limitation of the research data set, this analysis was proper in confirming the effectiveness of the weekly model.

### Daily Model (for Any Activity) Development and Performance

**Figure 5:**
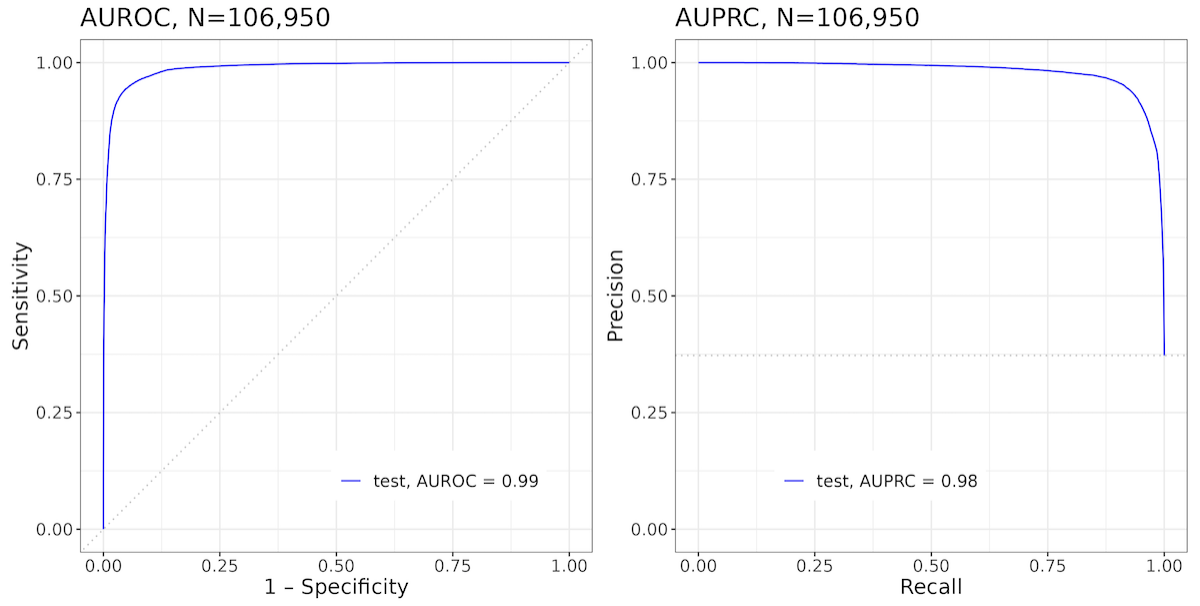
AUROC (left) and AUPRC (right) performance metrics of the “any activity” daily model in the test set of the daily vendor data set (106,950 engagement records). AUPRC: area under the precision-recall curve; AUROC: area under the receiver operating characteristic curve.

**Figure 6:**
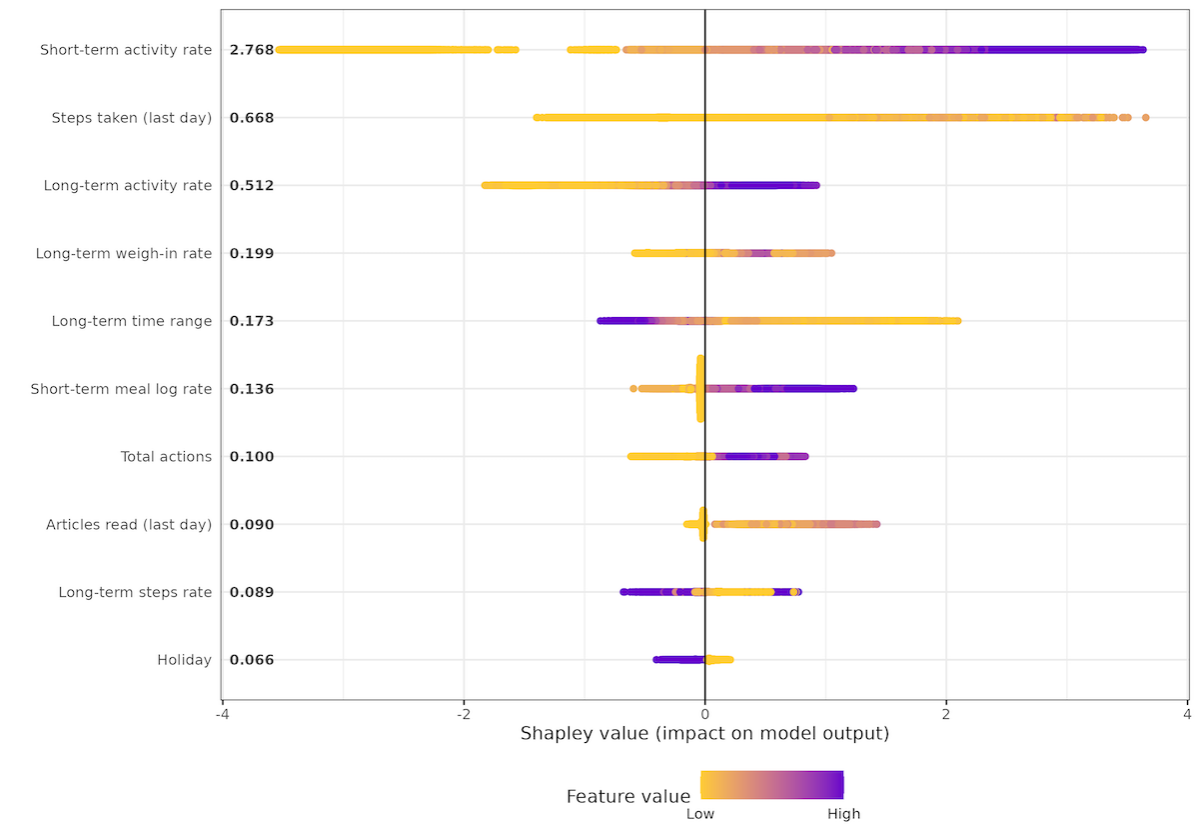
Shapley values of top 10 features in the “any activity” daily model. Each dot on the plot represents an engagement record and is colored according to the value of the corresponding feature from high (purple) to low (yellow). Features are ranked in descending order from top to bottom on the y-axis. Average Shapley values of each feature are annotated next to them on the y-axis.

We expanded a proportion of the weekly data set into a daily (more detailed) format and trained 5 new models. Figure 5 illustrates the ML model fit in the test set of the daily data set. Figure 6 displays the distribution of the covariates with the strongest predictive power (ie, the highest global mean Shapley value). Like the weekly model, engagement with any activity in the dDPP in the last 7 days had the most predictive power (a global mean Shapley value of 2.638). However, in contrast to the weekly model, features associated with long-term activity also had strong predictive power in the model.

Although the daily model for “any activity” returned a high AUROC and AUPRC, we aimed to generate predictions on each specific activity to inform our user profiling (digital engagement phenotypes) and consequently elevate the message personalization. Therefore, we developed 4 ML models, focusing on daily engagement with each key type of activity for a dDPP (physical activity, lessons, social activity, and weigh-ins). Table 2 displays the model fits for each of these “submodels.” For each activity, the model indicates highly predictive behavioral patterns among users. The “physical activity” and “social activity” daily models had higher AUROC performance with slightly lower AUPRC than the other daily models. All daily models show higher levels of calibration (a highest Brier score of 0.051) than the weekly model (a Brier score of 0.061).

**Table 2:** Performance metrics of each daily activity model in the test set.

Model fit metrics	Any app activity	Physical activity (exercises and steps)	Lessons (article reading)	Social activity (group posts and coach messages)	Weigh-ins
AUROC^a^ (95% CI)	0.99 (0.99-0.99)	0.98 (0.98-0.98)	0.99 (0.99-0.99)	0.98 (0.98-0.98)	0.94 (0.94-0.94)
AUPRC^b^ (95% CI)	0.98 (0.98-0.98)	0.74 (0.72-0.75)	0.91 (0.91-0.92)	0.74 (0.73-0.75)	0.65 (0.63-0.66)
Brier score (95% CI)	0.037 (0.036-0.038)	0.025 (0.025-0.026)	0.027 (0.026-0.028)	0.02 (0.023-0.024)	0.051 (0.050-0.052)

^a^AUROC: area under the receiver operating characteristic curve.

^b^AUPRC: area under the precision-recall curve.

### Engagement Profiling Development and Performance

We profiled participants with their daily engagement data using LPA after 2 weeks of dDPP enrollment. To determine the optimal time to start profiling participants, we iteratively added 1 day of engagement and created profiles until 3 weeks after their enrollment in the dDPP. After 2 weeks of daily engagement data, profiling participants had the strongest LPA model fit (BIC=−3222.46), followed by the model fit from profiling with 3 weeks of data (BIC=−2903.19). The LPA model fits for 15 to 20 days of engagement were significantly worse (ie, higher BIC values) and, therefore, are not reported. The best-performing LPA model was ellipsoidal (there is some correlation between variables), had equal volume (the variances are equal across identified profiles), had variable distributions between profiles (ie, the number of people per profile vary), and consisted of 6 profiles. Table 3 reports the mean engagement for each variable within and across the profiles of participants.

**Table 3:** Mean engagement by profile and across profiles for key engagement variables.

Key engagement variables	Subbehavior variable mean (SE)						Mean engagement across profiles (SD)
	Profile 1 (n=16)	Profile 2 (n=91)	Profile 3 (n=107)	Profile 4 (n=20)	Profile 5 (n=82)	Profile 6 (n=8)	
Any activity rate (last 7 days)	0.969 (0.085)	0.992 (0.045	1.000 (0)	0.747 (0.243)	0.115 (0.236)	0.814 (0.222)	0.752 (0.401)
Long-term activity rate	0.942 (0.105)	0.998 (0.013)	0.979 (0.049)	0.605 (0.262)	0.365 (0.292)	0.698 (0.244)	0.797 (0.318)
Steps taken rate (last 7 days)	2572 (4661)	3909 (3968)	3646 (3461)	1378 (1293)	0 (0)	1622 (2447)	2555 (3495)
Long-term weigh-in rate	0.507 (0.310)	0.139 (0.139)	0.265 (0.208)	0.0362 (0.043)	0.0471 (0.131)	0.241 (0.175)	0.172 (0.208)
Recent meal rate	0.906 (0.256)	0.397 (0.416)	0.690 (0.399)	0.0252 (0.112)	0.00305 (0.028)	0.00305 (0.297)	0.392 (0.444)
Long-term step rate	0.438 (0.345)	0.998 (0.013)	0.956 (0.068)	0.566 (0.292)	0.285 (0.295)	0.609 (0.303)	0.740 (0.359)
Long-term meal log rate	0.856 (0.249)	0.463 (0.339)	0.669 (0.349)	0.0543 (0.127)	0.0951 (0.145)	0.116 (0.076)	0.425 (0.386)
Article reading rate (last 7 days)	2.01 (1.549)	0.278 (0.704)	2.85 (1.644)	3.021 (0.923)	0 (0)	0.372 (1.061)	1.160 (1.689)

The LPA identified attrition (users in profile 5 who showed consistently low engagement across variables) and behaviors that show points of continued engagement for users. Users in profile 6, for example, had a close-to-average engagement with the dDPP from weigh-ins with the app and logging steps, which are behaviors that require one-time interactions with the dDPP, given Bluetooth connections between smart devices and the dDPP. In contrast, users in profile 3 were highly engaged, as they consistently engaged more than the average user. Messaging to users in profile 3 should, therefore, differ from messaging to users in profile 5, given the differences in their efforts toward the dDPP. Users in profile 4 had a lower-than-average engagement with the dDPP but showed the highest engagement with the learning materials across all users. Clusters 1 and 2 showed similarly high short- and long-term engagements but differed in engagement with the dDPP. Users in profile 1 read more educational materials provided in the dDPP, whereas users in profile 2 were more consistent in taking steps.

## Discussion

### Summary

The literature suggests the app of different ML algorithms to predict digital and traditional medication adherence and diverse intervention outcomes. Positive results of these studies support and validate the feasibility of applying ML methods to predict user engagement in digital health apps such as a dDPP to improve patient adherence to digital therapeutics and, consequently, health outcomes. In concordance with the literature, we applied the most suitable algorithm for our data set (gradient-boosted forest), yielded highly accurate results for predicting digital adherence, and identified variables with the strongest contribution to our outcome to understand digital behaviors [22-26]. This paper described 2 ML models developed using weekly and daily dDPP engagement data. First, using the weekly dDPP vendor data set, we developed a weekly ML model, which was validated using the collected data from this dDPP study. On the basis of past activity patterns, the model yielded high precision and recall and accurately predicted patient engagement for the next week. However, a model trained with weekly patient data can only predict weekly engagement, limiting our ability to gain detailed insight into a patient’s behavior. Because an ideal model should be robust to different dynamics in patients’ engagement data, we then developed a daily ML model using the daily dDPP vendor data set, which incorporates additional attributes, including the type of meals logged per day and calories. The daily model also yielded high precision and recall values. This finding supports using such models to anticipate behavior, focusing on identifying low engagement to intervene before attrition.

In addition to calculating precision and recall for our models, we calculated the Shapley values for both types of models (weekly and daily) to further analyze and identify which variables contribute the most to overall prediction. Results from the Shapley values revealed that short-term frequency of activity engagement was the most informative feature in the daily and weekly data analyses, meaning that users were more likely to form and stick to short-term behavioral patterns than long-term patterns in the dDPP. This finding is consistent with a previous study on predicting exercise and steps [27]. Because of user propensity to engage in short-term behaviors, we considered the daily model for individual activities best suited to develop engagement profiles. Using variables with high Shapley values from the daily model, we successfully created distinct digital engagement phenotypes of dDPP users. This allows for further research into developing infrastructure for tailored messaging to increase and maintain engagement with active users and intervene against attrition for inactive users. Specifically, identifying high engagement, minimal engagement, and attrition with early dDPP use lends itself to determining individuals facing barriers to dDPP engagement and improving dDPP implementation. Identifying strengths and weaknesses within behavior phenotypes through our profiling methods can also inform what specific behaviors (ie, low-engagement behaviors) need to be targeted in messaging for a user’s success in using the dDPP.

### Contributions and Implications

By leveraging digital behavioral usage data, we showed that we can successfully create digital engagement phenotypes, allowing for the future tailoring of digital health interventions based on patient needs. The methods used can extend beyond the prevention of metabolic disease, as an ML model incorporating behavioral usage variables can characterize prevention, maintenance, and wellness in other domains such as mental health, treatment adherence, and addiction prevention.

### Limitations

The weekly data sets posed limitations to maximizing patient engagement through integrating ML into PAMS. A model trained using weekly data is limited to predict weekly dDPP engagement (limited scope of dDPP engagement). The weekly ML model did not provide enough granularity to be robust to different dynamics of app engagement (eg, a sudden drop in engagement in 1 week due to vacation or a suddenly busy day where the user does not log information). The high sensitivity in a weekly engagement model to unexpected changes in usage could, therefore, negatively impact the type of messaging and timely motivation delivered to the patient. Consequently, we shifted the prediction cycle for engagement by moving from a model based on weekly behavior to one based on daily behavior.

Data showed that the short-term frequency of various activities was the most informative feature, but the results could mean that our model is vulnerable to short-term disruption of user behavioral patterns. Consequently, although the weekly data-based and daily data-based models were sufficient to prove the feasibility of using ML approaches for predicting patient engagement, further development is needed to refine these models and include extra patient information. Improvements include (1) understanding potential errors in the model and data sets (eg, data set size; using vendor data sets is an imperfect representation of other dDPP interventions) and (2) reviewing initial hypotheses about the data set and the choice of algorithms. To build the refined model, we would benefit from more detailed data. In this case, we would need to replan attributes and test other ML algorithms to perform further model improvements.

### Future Directions

With feasibility established, the next steps include creating user engagement phenotypes linked to personalized messaging interventions using behavior-based approaches to best motivate users to engage with the dDPP. We will also need to engineer the forest model and profile analysis to evolve as users change their engagement throughout participating in the dDPP so that messaging remains personalized to meet the users’ needs. Ultimately, this study demonstrated the potential value of ML and digital phenotyping to enhance the ability of digital behavior change interventions to predict engagement and personalize the interventions to maximize clinical impact.

## Abbreviations

AI: artificial intelligence
AUPRC: area under the precision-recall curve
AUROC: area under the receiver operating characteristic curve
BIC: Bayesian information criterion
dDPP: digital diabetes prevention program
DPP: diabetes prevention program
HIPAA: Health Insurance Portability and Accountability Act
IRB: institutional review board
LASSO: Least Absolute Shrinkage and Selection Operator
LPA: latent profile analysis
ML: machine learning
PAMS: personalized automatic messaging system
